# Interaction between phytoplankton and heterotrophic bacteria in Arctic fjords during the glacial melting season as revealed by eDNA metabarcoding

**DOI:** 10.1093/femsec/fiae059

**Published:** 2024-04-15

**Authors:** Dukki Han, Ki-Tae Park, Haryun Kim, Tae-Hoon Kim, Man-Ki Jeong, Seung-Il Nam

**Affiliations:** Department of Marine Molecular Bioscience, Gangneung-Wonju National University, Gangneung 25457, Republic of Korea; Korea Polar Research Institute, Incheon 21990, Republic of Korea; Department of Environmental Sciences and Biotechnology, Hallym University, Chuncheon 24252, Republic of Korea; East Sea Research Institute, Korea Institute of Ocean Science & Technology, Uljin 36315, Republic of Korea; Department of Oceanography, Faculty of Earth Systems and Environmental Sciences, Chonnam National University, Gwangju 61186, Republic of Korea; Department of Smart Fisheries Resources Management, Chonnam National University, Yeosu 59626, Republic of Korea; Korea Polar Research Institute, Incheon 21990, Republic of Korea

**Keywords:** Arctic glacial fjord, eDNA metabarcoding, heterotrophic bacteria, phycosphere, phytoplankton variability, rRNA

## Abstract

The hydrographic variability in the fjords of Svalbard significantly influences water mass properties, causing distinct patterns of microbial diversity and community composition between surface and subsurface layers. However, surveys on the phytoplankton-associated bacterial communities, pivotal to ecosystem functioning in Arctic fjords, are limited. This study investigated the interactions between phytoplankton and heterotrophic bacterial communities in Svalbard fjord waters through comprehensive eDNA metabarcoding with 16S and 18S rRNA genes. The 16S rRNA sequencing results revealed a homogenous community composition including a few dominant heterotrophic bacteria across fjord waters, whereas 18S rRNA results suggested a spatially diverse eukaryotic plankton distribution. The relative abundances of heterotrophic bacteria showed a depth-wise distribution. By contrast, the dominant phytoplankton populations exhibited variable distributions in surface waters. In the network model, the linkage of phytoplankton (Prasinophytae and Dinophyceae) to heterotrophic bacteria, particularly Actinobacteria, suggested the direct or indirect influence of bacterial contributions on the fate of phytoplankton-derived organic matter. Our prediction of the metabolic pathways for bacterial activity related to phytoplankton-derived organic matter suggested competitive advantages and symbiotic relationships between phytoplankton and heterotrophic bacteria. Our findings provide valuable insights into the response of phytoplankton-bacterial interactions to environmental changes in Arctic fjords.

## Teaser

This study investigates phytoplankton and bacteria relationships in Svalbard fjords, revealing spatial variations, potential ecological interactions and insights into how these interactions respond to environmental changes in Arctic fjords.

## Introduction

The Arctic glacial fjord in Svalbard is a unique and sensitive ecosystem experiencing rapid environmental changes owing to global warming (Svendsen et al. 2002). The distinctive characteristics of Arctic glacial fjords are expected to significantly impact the diversity of the Arctic marine ecosystem, encompassing various marine organisms from microscopic (eukaryotic phyto- and zooplankton and heterotrophic bacteria) to macroscopic (fish and benthic invertebrates) species. These organisms and with their interactions, are crucial in controlling the productivity and biodiversity of the Arctic fjord ecosystems (Hop et al. 2002). Particularly, the microbial food web in fjord ecosystems is primarily influenced by phytoplankton that supports primary production, and bacteria that transform phytoplankton-derived organic matter into inorganic nutrients (Rokkan Iversen and Seuthe 2011, Buchan et al. 2014).

The interactions between phytoplankton and bacteria, which are closely associated in the microbial food web, have been postulated for decades (Seymour et al. 2017). Phytoplankton, such as diatoms (eukaryotes), dinoflagellates (eukaryotes) and cyanobacteria (prokaryotes), are responsible for almost 50% of global photosynthesis in aquatic environments (Falkowski 1994, Field et al. 1998). Furthermore, these phototrophic organisms are consequently linked to biogeochemical cycles mediated by heterotrophic bacterial metabolism (Seymour et al. 2017). Emerging evidence indicates that the interaction of phytoplankton and bacteria at the base of the food web controls carbon and nutrient cycling in oceans (Seymour et al. 2017). However, its ecological interface in Arctic fjords remains uncertain.

Phytoplankton communities in the Arctic Svalbard have been extensively surveyed under microscopic observation (Piwosz et al. 2009, Hodal et al. 2012, Marquardt et al. 2016, Caroppo et al. 2017, Hegseth et al. 2019, Payne and Roesler 2019, Bae et al. 2022, Kohlbach et al. 2023). Recent observations suggested that summer fjord waters may not be conducive to the survival of eukaryotic phytoplankton, such as diatoms or dinoflagellates, because of nutrient limitations and salinity changes resulting from glacial melting (Bae et al. 2022). Unlike other oceans, underwater light conditions are determined by the thickness of sea ice and its snow cover in the Arctic Ocean; these conditions control the formation of early phytoplankton assemblages. Additionally, the post-phytoplankton bloom in summer Arctic fjords is limited because of the reduced light transparency resulting from increased sediment-laden waters after glacial melt discharge (Wiktor 1999). Similarly, bacterial surveys in Arctic fjords have revealed that Arctic hydrography affects the bacterial community composition in the Svalbard fjords (Zeng et al. 2009, Piquet et al. 2016, Jain and Krishnan 2017, Han et al. 2021, Han et al. 2022b); this implies a role for bacterial degradation of organic matter produced and released by phytoplankton (Han et al. 2021, 2022). However, phytoplankton-associated bacterial communities have been studied less frequently in Arctic fjords than in other environments. This information can explain how inorganic nutrients are resupplied to primary producers through organic matter degradation in Arctic fjords.

A phytoplankton cell conserves its immediate microenvironment (phycosphere). It is a unique and dynamic niche where the cell continuously releases organic molecules into the surrounding water, including the bacterial habitat (Seymour et al. 2017). Phytoplankton-derived organic matter, including polyphenols, alkaloids, terpenes, polysaccharides, fatty acids, sterols, lactones, proteins and peptides, significantly contributes to the resupply of nutrients to primary producers through bacterial degradation (Bhowmick et al. 2020). Thus, understanding the fate of phytoplankton-derived organic matter in Arctic glacial fjords is essential to gain insights into the ecological and biogeochemical processes in this unique region and to predict how bacterial responses to future environmental changes in the Arctic ecosystem will impact these processes.

Although the investigation of the phytoplankton-bacterial interface has been challenging because of its complex and dynamic nature, recent advances in molecular techniques have provided new tools to investigate the phytoplankton and bacterial communities, including the use of environmental DNA (eDNA) metabarcoding. eDNA metabarcoding has emerged as a powerful tool for biodiversity assessment in various environments (Yoccoz 2012, Deiner et al. 2017). This technique involves amplifying and sequencing a specific DNA marker from environmental samples to identify the organisms in a community. Compared with other conventional methods, eDNA metabarcoding is a more efficient and cost-effective approach for assessing the composition and diversity of biological communities (Pompanon et al. 2012, De Barba et al. 2014). Recent studies have employed eDNA metabarcoding to investigate the bacterial and phytoplankton communities in marine environments using 16S (Agogué et al. 2011, Ghiglione et al. 2012, Han et al. 2014, 2015, Techtmann et al. 2015, Hernando‐Morales et al. 2017) or 18S (Logares et al. 2012, De Vargas et al. 2015, De Luca et al. 2021, Šupraha et al. 2022, Cui et al. 2023) rRNA genes, respectively. Moreover, comprehensive 16S and 18S rRNA community analyses have validated the interactions between phytoplankton and bacterial communities on the southern coast of the Korean Peninsula (Han et al. 2022a) and in the coastal ocean of southern California (Needham et al. 2018). However, no study has investigated the phytoplankton-bacteria interface using the eDNA metabarcoding approach in Arctic fjords. To bridge this gap, in this study, we used eDNA metabarcoding to investigate the bacterial communities associated with phytoplankton in the Svalbard Arctic fjord. A diverse and distinct community was revealed in response to dynamic environmental conditions during the summer melting season in four different Arctic fjords (van Mijenfjorden, van Keulenfjorden, Hornsund and Storfjorden).

## Materials and methods

### Description of sample preparation and datasets

**Figure 1.**
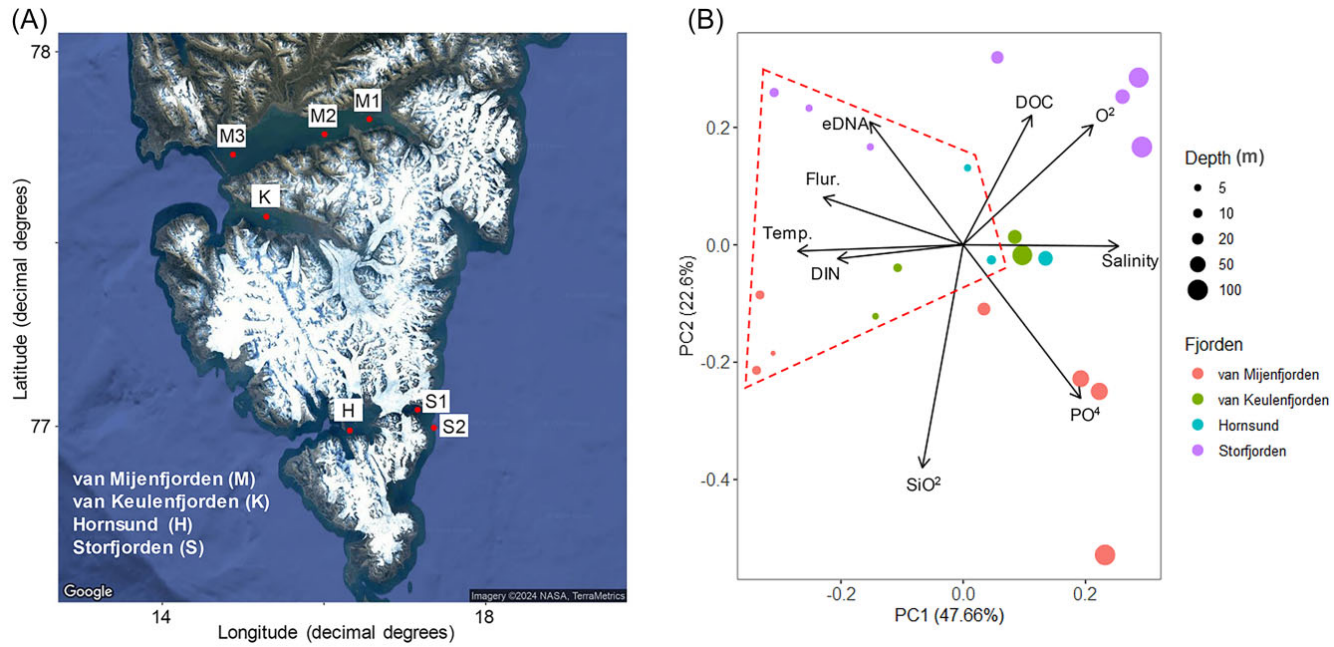
Description of research area and samples in the Svalbard. (A) Sampled locations and (B) water mass properties of sampled waters visualized by principal component analysis (PCA). The geographic locations of the sampled area in Svalbard were visualized using the ggmap package (Kahle et al. 2019) in R. In PCA, samples enclosed with red colored dashed lines indicate surface waters (shallower than 10-m depth).

The survey of Arctic Svalbard fjords (van Mijenfjorden, van Keulenfjorden, Hornsund and Storfjorden) was conducted using R/V Helmer Hanssen in August 2019 (Fig. 1A). Seawater samples (*n* = 21, each 2 L of seawater) and hydrographic data (fluorescence, oxygen, salinity and temperature) were collected from the fjords at discrete depths using a shipboard conductivity-temperature-depth rosette system (Seabird SBE 911plus; Sea-Bird Electronics, Bellevue, USA). Sampled depths are provided in the supplementary Excel data and indicated in the sample IDs. Each 1 L of sampled seawater was immediately filtered in the field using a 0.2-µm hydrophilic PVDF membrane (Merck, Darmstadt, Germany) and the filtered membranes were then stored in a deep freezer (−80°C) until eDNA extraction. eDNA was extracted from two membrane filters at each sampled depth using a DNeasy PowerWater Kit (QIAGEN, Hilden, Germany) following the manufacturer's instructions and its quantity was estimated using a Qubit 4.0 Fluorometer (Invitrogen, CA, USA). Next, 50 ml of seawater were additionally filtered (0.45-µm HDPE syringe filter) and frozen (−20°C) until nutrient measurement for dissolved inorganic nitrogen (DIN: NO_3_^−^ + NH_4_^+^), phosphate (PO_4_^+3^) and silicate (SiO_2_^−^) using a nutrient auto-analyzer (QuAAtro 39; Seal Analytical Inc., Mequon, USA). For dimethylsulphoniopropionate (DMSP) analysis, 20 ml of seawater was collected in an amber vial, acidified to pH < 2 by adding 100 µL of 50% H_2_SO_4_, and subsequently stored at 4°C until analysis. The preserved DMSP sample was hydrolyzed to gaseous dimethyl sulfide with 10 M NaOH (addition of 0.25 ml per mL of the sample). The evolved dimethyl sulfide was then quantified using gas chromatography with an attached flame photometric detector (Agilent 6890 N; Agilent Technologies, Wilmington, USA) (Park et al. 2014).

This study compiled the datasets into 21 samples of 16S and 18S rRNA gene sequences and their metadata. 18S rRNA gene sequences were constructed from this study, and 16S rRNA sequences were selectively retrieved from the National Center for Biotechnology Information (NCBI) under accession number PRJNA669167. The metadata for these sequences constituted the hydrographic measurements, nutrients and dissolved organic carbon (DOC), retrieved from the previous study (Han et al. 2022b).

### eDNA metabarcoding and ribosomal gene quantification

A metabarcoding-based approach was applied to sequence the rRNA hypervariable region of eDNAs using an Illumina MiSeq platform (Macrogen, Seoul, South Korea). The eDNA metabarcoding with two-step PCRs amplified the 16S and 18S rRNA genes to analyze bacterial and eukaryotic communities. Information on used primer sequences and detailed conditions of the two-step PCRs were previously described (Han et al. 2022a). Briefly, the first amplicon PCRs were carried out in triplicate using a KAPA HiFi Hotstart ReadyMix PCR kit (KAPA BioSystems, MA, USA) with sequencing primers for 16S (V3–V4) and 18S (V8–V9) rRNA genes. The PCR amplicons were purified using a Qiaquick PCR purification kit (Qiagen, CA, USA), and then the second index PCRs proceeded using the PCR amplicons according to Illumina's instructions. Concentrations of the index PCRs were measured by the Qubit 4.0 Fluorometer (Invitrogen, CA, USA) after amplicon purification. The purified amplicons were all mixed in equimolar amounts to construct the Illumina MiSeq library and subjected to sequencing. The obtained metabarcoding sequences were submitted to the Sequence Read Archive of the NCBI under the accession number PRJNA669202 (eukaryotic 18S rRNA gene).

Quantification of 16S and 18S rRNA genes was carried out in triplicate using a real-time quantitative PCR (qPCR) with a qSYBR Premix Ex Taq™ (Takara Bio Inc., Shiga, Japan). Each content of rRNA genes was measured using bacterial specific primer (Lane 1991) (Bac338f: 5′-ACTCCTACGGGAGGCAGC-3′; Bac518r: 5′-ATTACCGCGGCTGCTGG-3′) and eukaryotic specific primer (Zhu et al. 2005) (EUK345f: 5′-AAGGAAGGCAGCAGGCG-3′; EUK499r: 5′-CACCAGACTTGCCCTCYAAT-3′) sets. The quantification standard for the threshold cycle (Ct) calibration of a target gene constitutes a dilution series of a known amount of genomic DNA (gDNA) for *Escherichia coli* ATCC 11775 and *Yarrowia lipolytica* ATCC 20306. qPCRs for the target genes in samples and standards were performed with the following conditions: initial denaturation at 94°C for 3 min, followed by 40 cycles of denaturation at 94°C for 20 s, specific annealing of each target gene at 60°C for 30 s and elongation at 72°C for 30 s. At the end of each run, a dissociation melt curve of the PCR product was determined to verify amplicon specificity. The copy number of 16S and 18S rRNA genes was calculated using the following formula with putative gDNA of *E. coli* (5 038 133 bp genome and seven copies of 16S rRNA gene) (Meier-Kolthoff et al. 2014) and *Y. lipolytica* (20 262 281 bp genome and 182 copies of 18S rRNA gene) (Tejerizo et al. 2017), respectively. rRNA genes per nanogram gDNA = [6.022 × 10^23^ (Avogadro's constant) / (base pairs of genome × 660 (average mass of 1 bp of dsDNA) × 10^9^ (conversion factor)] × number of rRNA genes per genome).

### Sequence data processing and analysis

Sequencing data analysis was performed using the Mothur software package (v. 1.40.5) (Schloss et al. 2009) based on the MiSeq standard operating procedure (SOP) (Kozich et al. 2013). Briefly, 5 699 514 sequences for the 16S rRNA and 2 523 682 sequences for the 18S rRNA were obtained. The process of sequence quality filtration commenced by correcting amplification and sequencing errors, followed by singleton removal and random subsampling of sequences using the given commands in Mothur SOP. Before the random sampling, non-bacterial sequences like chloroplasts, mitochondria and unknown were removed using the “remove.lineage” command after taxonomic classification using the “classify.seqs” command. The sequence count number for each sample was normalized with 10 000 sequences of both 16S and 18S rRNA genes in the random subsampling. These filtered sequences were clustered into the amplicon sequence variants (ASVs) to investigate microbial alpha and beta diversity and community composition. In the Mothur Miseq SOP, abundance-unweighted species richness indices (Chao1 and ACE) were calculated using ASVs for the alpha diversity, and a phylip-formatted distance matrix using thetayc dissimilarity was generated for the beta diversity. Non-metric multidimensional scaling was applied to visualize beta diversity, and its statistical separation was supported by analysis of molecular variance. The composition of the microbial community was determined against the Silva.seed_v132 database.

Statistical analysis was performed using R software (v. 4.2.0) (https://www.R-project.org) with various R packages. Principal component analysis (PCA) and simple linear regression analysis were carried out using the function “prcomp” and “stats” of the package “stats” (R core team 2017). Multi-response permutation procedure (MRPP) and indicator species analysis were carried out using the “mrpp” function of the vegan package (Oksanen and Blanchet 2017) and the “indval” function of the labdsv package (Roberts and Roberts 2016), respectively. Microbial network interactions with environmental factors were estimated using the RCy3 package (Gustavsen et al. 2019) connected to the Cytoscape software (v. 3.10.0) (https://cytoscape.org). The network analysis proceeded with sequences assigned to ASVs or class-level taxonomy. Additional data curation was performed to minimize the potential inaccuracies caused by a significant number of zero values in the ASVs dataset. In the initial stage, 17 861 bacterial ASVs and 4376 eukaryotic ASVs, all with a sequence count exceeding 10, were filtered from the dataset. Subsequently, major ASVs were selected based on each ASV representing more than 0.1% of the total dataset. Using the curated ASV dataset, the network model was similarly constructed following the procedures used for the class-level taxonomy dataset. Spearman's correlation matrix was calculated by the relative abundance of bacterial and eukaryotic populations (class level) and values of environmental factors. Co-occurrence patterns between microbial populations or between population and environmental factors were determined by significant coefficient values more than 0.5 (*P* < 0.05) in Spearman's correlation and were visualized in network interactions. The functional potential of bacterial communities was estimated using the software PICRUSt2 (Douglas et al. 2020). Predictive functional abundance tables were classified using the Kyoto Encyclopedia of Genes and Genomes (KEGG). The abundance of KEGG data was displayed by a heatmap visualization using the “HeatmapAnnotation” function of the ComplexHeatmap package (Gu et al. 2016).

## Results

### Water mass properties of Svalbard fjords

The water samples collected from distinct depths in the Svalbard fjords (Fig. 1A and Table 1: van Mijenfjorden (n = 7), van Keulenfjorden (n = 4), K1–K2, Hornsund (n = 3) and Storfjorden (n = 7)) were explained by their water mass properties in the PCA patterns (Fig. 1B). The proportion of variance in the PCA ordination was represented by two axes: PC1 (47.66%) and PC2 (22.60%). Among the water mass properties, temperature and salinity primarily defined the PC1 axis, whereas silicates mostly explained the PC2 axis. Water mass properties on the PCA revealed that surface (≤10-m depth) waters were separated from subsurface (>10 m) waters on the PC1 axis, and the significance of their separation was supported by MRPP (*P* < 0.05). The separation between the surface and subsurface waters was visualized using temperature and salinity diagrams (Fig. S1). Moreover, the PC1 axis suggested an increase in salinity and a decrease in temperature, DIN and chlorophyll fluorescence during the transition of water masses from the surface to subsurface layers. Indeed, DIN and chlorophyll fluorescence were higher in the surface waters than in the subsurface waters, and their variations revealed significantly stronger correlations with depth (Fig. S2). Contrastingly, the PC2 axis suggested spatially distinguishable waters with variations in silicate and DOC in van Mijenfjorden and Storfjorden. For example, the silicate concentration in van Mijenfjorden was higher than that in Storfjorden; however, the DOC values showed the opposite pattern (Fig. S3). eDNA concentrations varied among fjord waters but showed no depth-wise distribution (Fig. S4). Despite such eDNA variation, the copy numbers of 16S and 18S rRNA genes showed a relatively conserved distribution among the fjord waters (Fig. S4). DMSP concentrations were estimated using Pearson's correlation, in conjunction with simple linear regression analysis, to analyze their relationship with changes in depth (Fig. S5). The concentration of DMSP demonstrated a significant negative correlation with depth (r: −0.73, R^2^: 0.48, *P* < 0.05), manifesting higher values in surface waters (11.51 ± 9.97 nM) than in subsurface waters (1.31 ± 0.47 nM).

**Table 1.** Sample description. Detailed information on associated metadata for sequenced samples is provided in the supplementary data.

ID	Fjord	Latitude (N)	Longitude (E)	Bottom depth (m)	Sampled depth (m)	Metabarcoding target gene (data source)	
M1_9	van Mijenfjorden	77.82 593	16.5598	68	9	16S rRNA V3-V4 region (Han et al. 2022b)	18S rRNA V8-V9 region (this study)
M1_28					28		
M1_63					63		
M2_3		77.78663	16.00682	73	3		
M2_9					9		
M3_60		77.7334	14.87518	107	60		
M3_102					102		
K_4	van Keulenfjorden	77.56957	15.28742	99	4		
K_8					8		
K_35					35		
K_93					93		
H_5	Hornsund	76.9886	16.31862	115	5		
H_10					10		
H_40					40		
S1_5	Storfjorden	77.04595	17.15692	98	5		
S1_10					10		
S1_40					40		
S1_95					95		
S2_5		76.99618	17.35718	112	5		
S2_25					25		
S2_105					105		

### Diversity and community composition of 16S and 18S rRNA gene sequences

**Figure 2.**
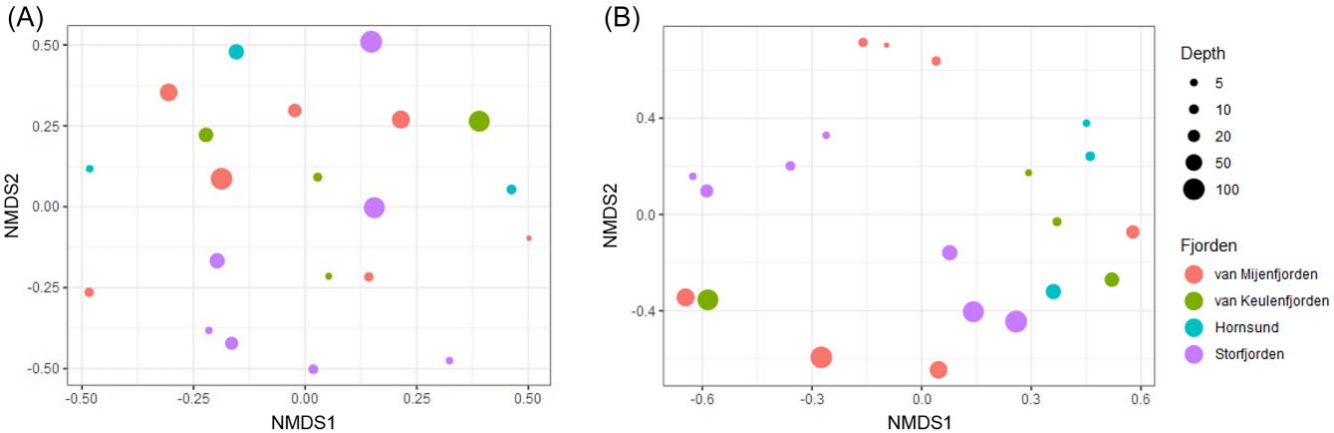
Beta diversity patterns for (A) 16S and (B) 18S rRNA gene metabarcoding data visualized by non-metric multidimensional scaling (NMDS).

Each of the 210 000 sequences of the 16S and 18S rRNA genes was clustered at 17 861 and 4378 ASVs, respectively. These ASVs were analyzed for alpha and beta diversity. The alpha diversity indices (Chao1 and ACE) of both 16S and 18S rRNA sequences were not significantly correlated with depth change (ρ < 0.5, *P* > 0.01) but showed more variability in 18S rRNA than in 16S rRNA (Fig. S6). The less variable Chao1 and ACE indices of 16S rRNA suggested spatially similar species richness of bacterial communities among the fjord waters. The 18S rRNA indices were relatively lower in van Mijenfjorden and Storfjorden than in the other fjords, indicating a higher species richness of eukaryotes in van Keulenfjorden and Hornsund. Further, the beta diversity in 16S and 18S rRNA on non-metric multidimensional scaling revealed separate clusters between the surface and subsurface waters (Fig. 2), and this separation was significantly supported by analysis of molecular variance (*P* < 0.01; Table S1).

**Figure 3.**
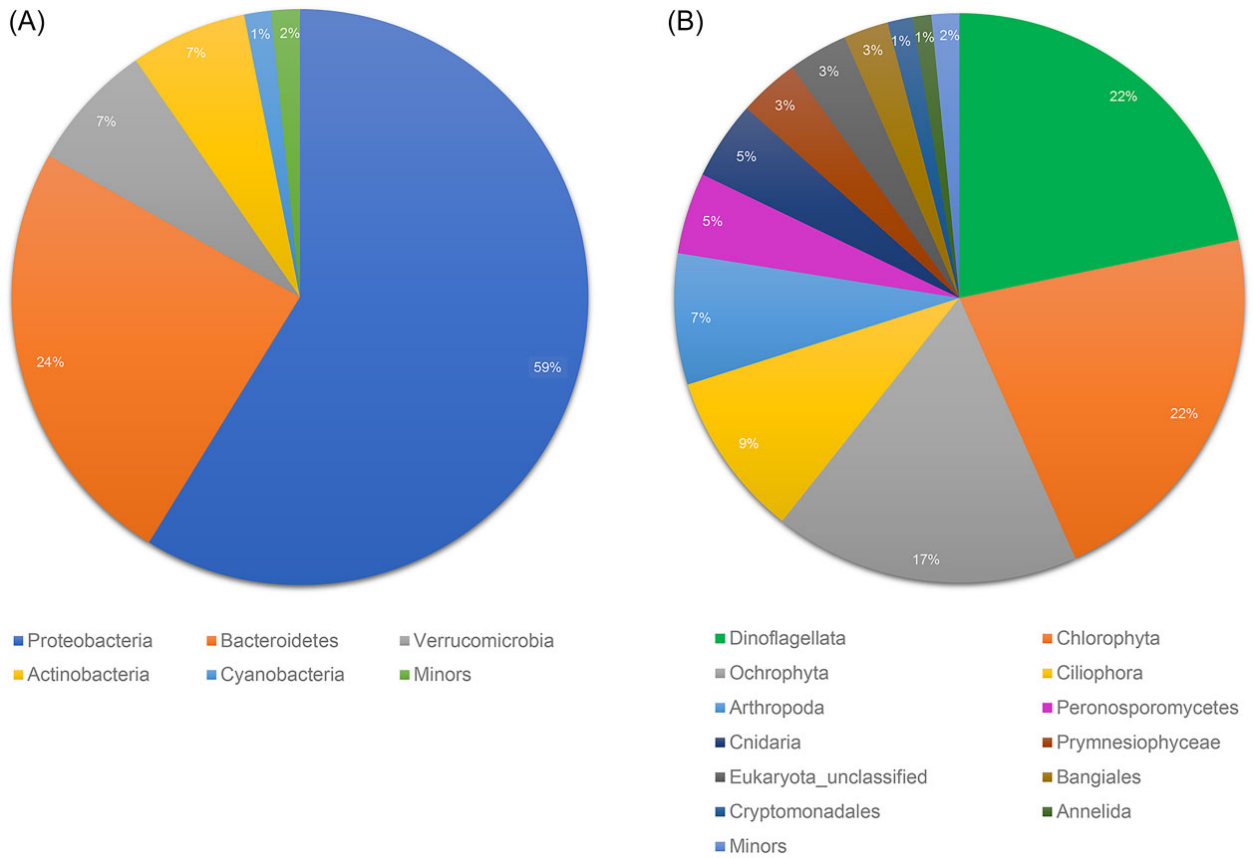
Taxonomic classification of (A) 16S and (B) 18S rRNA data at phylum level.

Using the taxonomy database, 420 000 sequences of 16S and 18S rRNA genes were classified. The 16S rRNA sequences were assigned to bacterial populations, such as Proteobacteria (59%), Bacteroidetes (24%), Verrucomicrobia (7%), Actinobacteria (7%) and Cyanobacteria (1%), and most 18S rRNA sequences were identified as eukaryotic phytoplankton (68%; Dinoflagellata, Chlorophyta, Ochrophyta, Prymnesiophyceae, Bangiales and Cryptomonadales) and zooplankton (22%; Ciliophora, Arthropoda, Cnidaria and Annelida) (Fig. 3). At the phylum level, these taxa represented spatially distinguishable variation between bacterial and eukaryotic communities (Fig. S7). For example, Proteobacteria, Bacteroidetes and Actinobacteria were dominant in all fjord waters, and their relative abundances revealed a ubiquitous distribution among the fjords. However, surface waters harbor relatively abundant cyanobacterial sequences and limited sequences of Verrucomicrobia. In contrast to bacterial communities, eukaryotic populations are variably distributed among the fjord waters. The van Mijenfjorden and Storfjorden waters exhibited remarkably distinguishable distributions of eukaryotic phytoplankton (Dinoflagellata, Chlorophyta and Ochrophyta).

**Figure 4.**
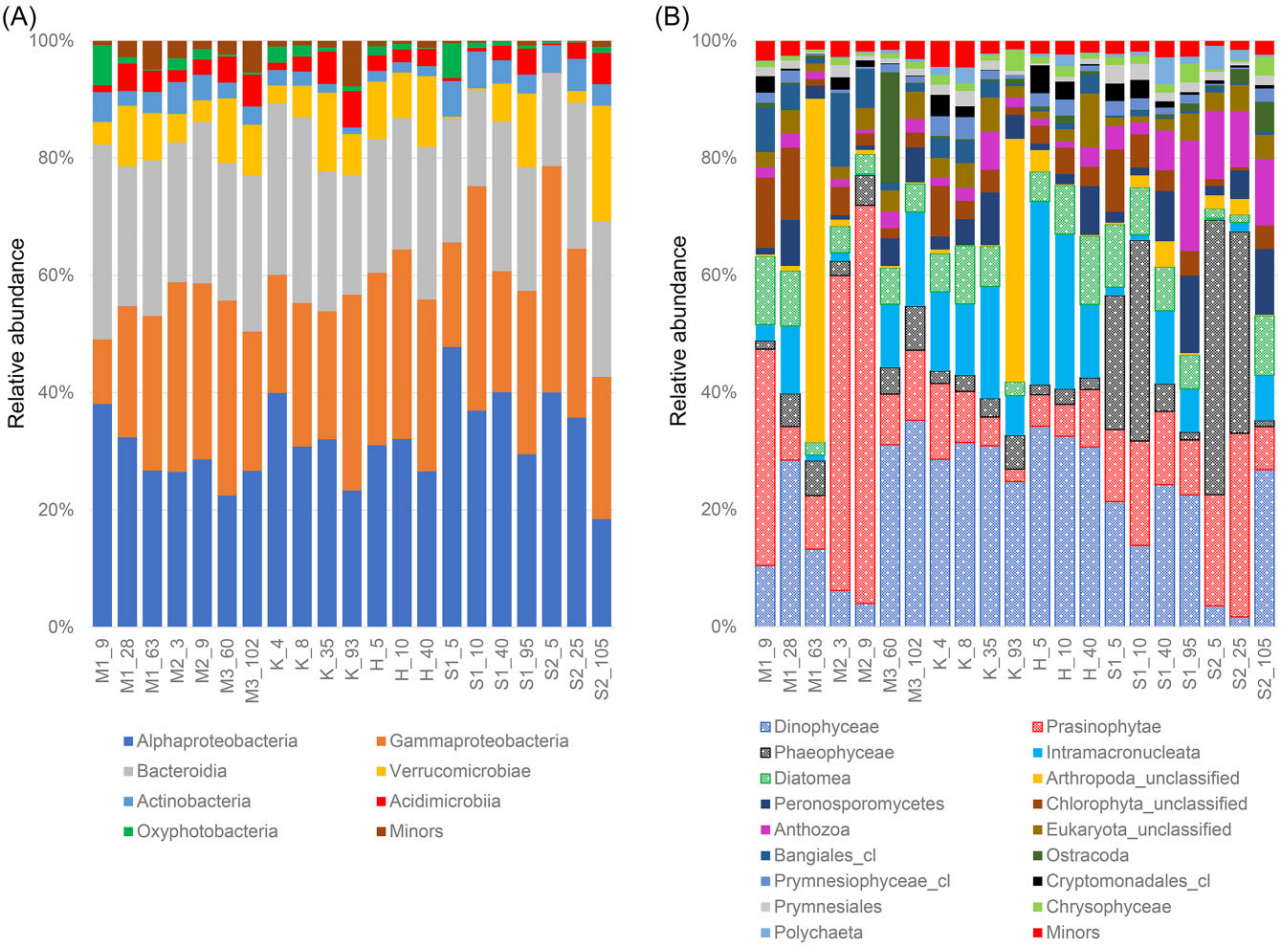
Composition of (A) bacterial and (B) eukaryotic communities at class level.

Such distributions of bacterial and eukaryotic communities were more distinctly visualized at the taxonomic class level. Heterotrophic bacteria, such as Alphaproteobacteria, Gammaproteobacteria and Bacteroidia, were dominant among the samples, and their relative abundance showed a depth-wise distribution. Still, the relative abundance of photosynthetic Oxyphotobacteria was limited to the surface waters (Fig. 4A). Other photosynthetic populations in the eukaryotic communities revealed spatially distributed patterns in the fjord waters (Fig. 4B). For example, Dinophyceae was the most predominant photosynthetic player in both surface and subsurface waters from van Keulenfjorden and Hornsund, and in subsurface waters from van Mijenfjorden and Storfjorden. Prasinophytae was dominant in the surface waters of van Mijenfjorden, whereas Phaeophyceae was predominantly found in the surface waters of Storfjorden. Contrastingly, Diatomea was not dominant compared with other phytoplankton, but was more evenly distributed in the fjord waters. In the case of zooplankton, Intramacronucleata was abundant in van Keulenfjorden and Hornsund, and its spatial variation was like that of Dinophyceae. Anthocyanins were relatively dominant in Storfjorden, but other zooplankton (Arthropoda_unclassified or Ostracoda) occasionally occurred in some subsurface waters. At the genus level of taxonomy, the structure of both bacterial and eukaryotic communities was complex, with a considerable number of taxa remaining taxonomically unclassified (Fig. S8). Despite this, the distribution of the most predominant taxa at the class level was reflected at the genus level. For example, the distributions of two cold-adapted genera, SAR11 clade Ia (Kraemer et al. 2020) and *Psychrobacter* (Rodrigues et al. 2009), represented the prevalence of Alphaproteobacteria and Gammaproteobacteria, respectively, within the bacterial community. In particular, the relative abundance of SAR11 clade Ia appeared to gradually decrease from surface to subsurface waters. Similarly, the most dominant eukaryotic genera identified in this study, *Pterosperma* of class Prasinophytae and *Prorocentrum* of class Dinophyceae, which have previously been recorded in Arctic coastal waters (Sala-Pérez et al. 2016, Joli et al. 2017), exhibited spatial distribution patterns that closely matched those of Prasinophytae and Dinophyceae, respectively.

Bacterial and eukaryotic populations at the taxonomic class level were used for indicator species analysis (Table S2). Although none of the significant representatives for fjord-specific populations (indicator value < 0.6) were determined by indicator species analysis, five representative populations for surface or subsurface water had significant indicator values (>0.7, *P* < 0.01) (Table 2). In bacterial populations, the prokaryotic phytoplankton, Oxyphotobacteria, specifically occurred in the fjord surface waters, but heterotrophic bacteria, such as Acidimicrobiia and Verrucomicrobiae, were representative of the subsurface waters. Like bacterial populations, Cryptomonadales_cl (eukaryotic phytoplankton) and Peronosporomycetes (heterotrophic eukaryotes) represented the surface and subsurface waters, respectively. These microbial population dynamics in the Svalbard fjords implied the potential role of photosynthetic (surface) and heterotrophic (subsurface) players in the planktonic food web.

**Table 2.** Indicator species analysis for 16S and 18S rRNA data in surface or subsurface water groups. The highest indicator values with significance (*P* < 0.01) are highlighted in bold and underlined.

rRNA	Taxa (class)	Group (depth)	Indicator value	*P* value
16S	Oxyphotobacteria	Surface	**0.82**	**≤0.01**
	Actinobacteria		0.58	>0.01
	Alphaproteobacteria		0.55	>0.01
	Gammaproteobacteria		0.51	>0.01
	Acidimicrobiia	Subsurface	**0.74**	**≤0.01**
	Verrucomicrobiae		**0.72**	**≤0.01**
	Bacteroidia		0.5	>0.01
18S	Cryptomonadales_cl	Surface	**0.89**	**≤0.01**
	Bangiales_cl		0.72	>0.01
	Prasinophytae		0.7	>0.01
	Phaeophyceae		0.64	>0.01
	Prymnesiophyceae_cl		0.62	>0.01
	Chlorophyta_unclassified		0.61	>0.01
	Prymnesiales		0.61	>0.01
	Polychaeta		0.59	>0.01
	Diatomea		0.53	>0.01
	Arthropoda_unclassified	Subsurface	0.89	>0.01
	Ostracoda		0.88	>0.01
	Peronosporomycetes		**0.81**	**≤0.01**
	Anthozoa		0.69	>0.01
	Eukaryota_unclassified		0.63	>0.01
	Dinophyceae		0.57	>0.01
	Chrysophyceae		0.56	>0.01
	Intramacronucleata		0.52	>0.01

### Network analysis of the microbial populations

**Figure 5.**
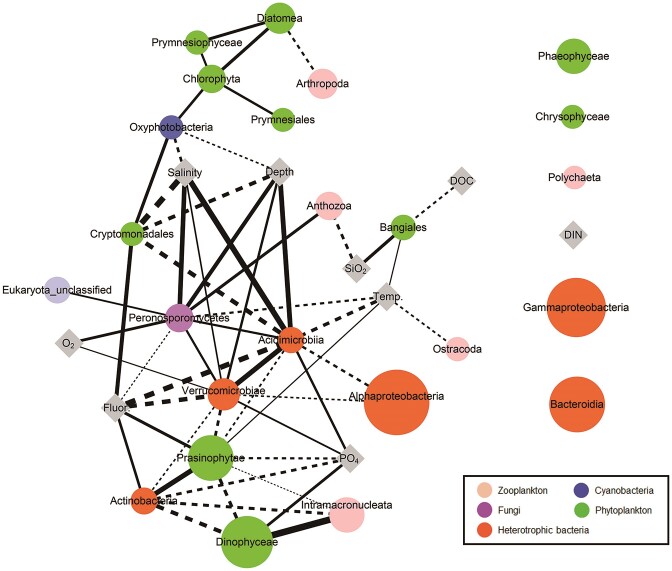
Microbial network model with water mass properties. In the network model, each node indicates microbial populations (pale pink circle: zooplankton; dark blue circle: cyanobacteria; purple circle: fungi; green circle: phytoplankton; orange circle: heterotrophic bacteria) or water mass properties (gray diamond). The size of microbial nodes and edge between nodes indicate their relative abundance and correlation, respectively. Thickness of edge represents a correlation coefficient (the thicker the line, the stronger correlation), and the solid lines and dotted lines represent positive and negative correlations, respectively. The isolated nodes with no edge indicate there was no interaction at a significant level (correlation coefficient < 0.5, *P* > 0.05).

The network analysis provided insights into the dynamics of microbial populations by analyzing major ASVs (each representing >0.1% of the total dataset) through Spearman's correlation at a significant level (ρ > 0.50, *P* < 0.05). Evidently, nodes determined by ASVs of heterobacteria surrounded the ASVs of autotrophic bacteria and other eukaryotes, including phytoplankton, zooplankton, fungi and unclassified eukaryote. To extract specific interactions among these populations, microbial population dynamics at the class level with water mass properties were visualized in the network analysis (Fig. 5), wherein microbial co-occurrences and their interactions with the water mass properties were evaluated using Spearman's correlation. Network co-occurrence consisted of most microbial taxa with water mass properties at a significantly strong correlation level (ρ > 0.59, *P* < 0.05), except for those of two bacterial (Gammaproteobacteria and Bacteroidia) and three eukaryotic (Phaeophyceae, Chrysophyceae and Polychaeta) populations and dissolved organic nitrogen. In the microbial network, four phytoplankton groups (Chlorophyta, Diatomea, Prymnesiales and Prymnesiophyceae) were positively correlated without any interactions with water mass properties. Other phytoplankton, such as Bangiales, Cryptomonadales, Dinophyceae and Prasinophytae, interacted more with heterotrophic populations or water mass properties. Photosynthetic cyanobacteria (Oxyphotobacteria) were positively correlated with other phytoplankton groups (Chlorophyta and Cryptomonadales) and negatively correlated with salinity and depth. Contrastingly, heterotrophic bacteria (Acidimicrobiia, Actinobacteria and Verrucomicrobiae) were diversely connected to the network components and two zooplankton (Arthropoda and Anthozoa) were negatively connected to Diatomea and silicate, respectively.

### Prediction of bacterial metabolisms

**Figure 6.**
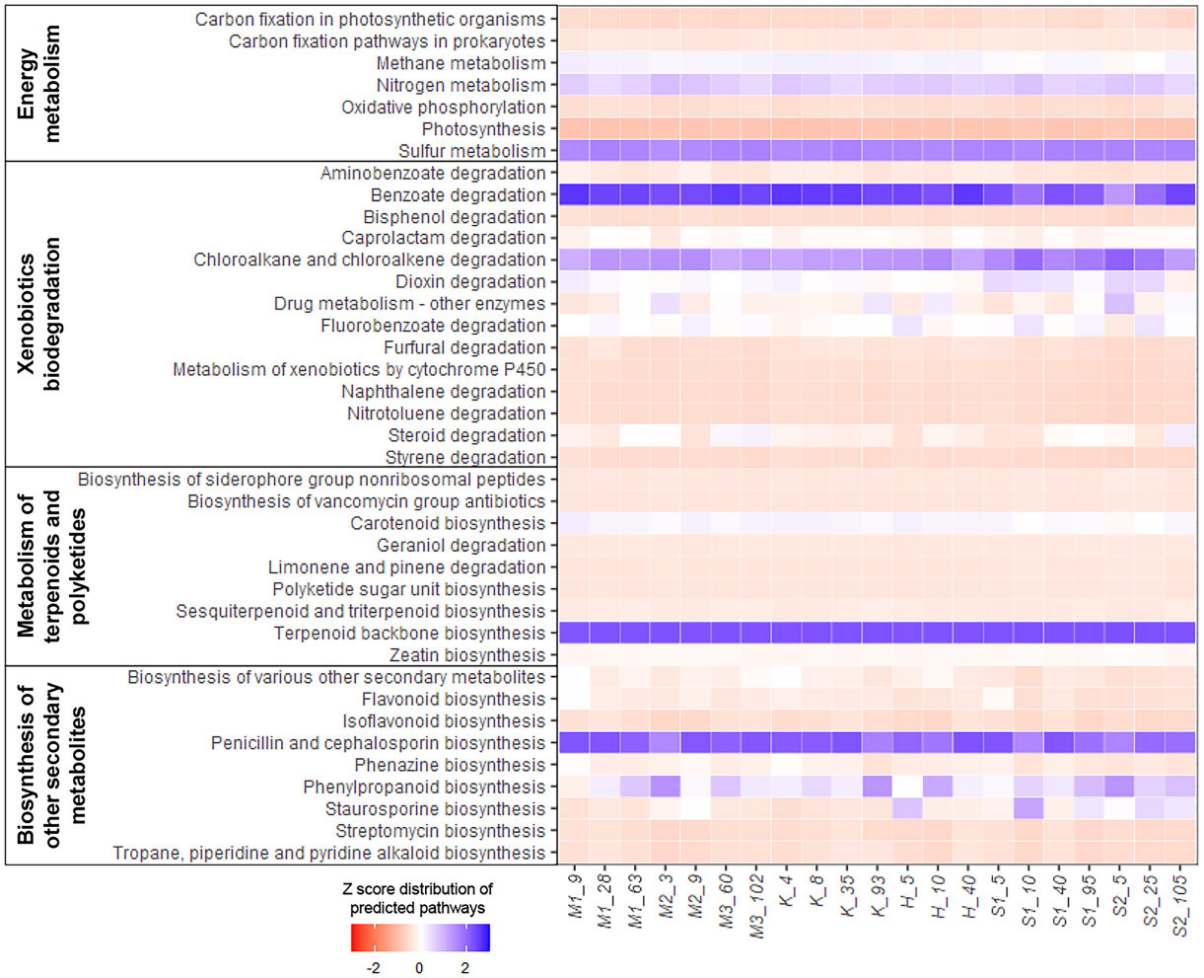
Prediction of metabolic pathways for bacterial activity in energy metabolism, xenobiotic biodegradation, metabolism of terpenoids and polyketides, and biosynthesis of secondary metabolites.

Bacterial metabolism predictions were conducted using PICRUSt2, based on ASVs derived from 16S rRNA sequences. In total, 17 861 ASVs were identified from 21 000 sequences. Rare ASVs with <10 sequences were excluded to optimize the use of limited computing resources, resulting in 1874 ASVs, corresponding to 85.13% of the total sequence dataset. Following the exclusion of rare ASVs, the metabolic prediction identified predominant pathways related to the metabolism of carbohydrates, amino acids, cofactors, vitamins and nucleotides within the fjord waters (Fig. S10). We investigated the pathways associated with energy metabolism, xenobiotic biodegradation, terpenoid and polyketide metabolism, and secondary metabolite biosynthesis (Figs 6 and S11). Relatively featured pathways in each metabolism were found (energy metabolism: sulfur metabolism (36.31%) and nitrogen metabolism (23.06%); xenobiotics biodegradation: benzoate degradation (37.55%) and chloroalkane and chloroalkene degradation (25.35%); metabolism of terpenoids and polyketides: terpenoid backbone biosynthesis (74.79%); biosynthesis of other secondary metabolites: penicillin and cephalosporin biosynthesis (52.27%) and phenylpropanoid biosynthesis (22.44%)).

## Discussion

The regional hydrographic variability of Svalbard is primarily influenced by two main currents (Arctic and Atlantic waters), and their hydrographic balance with glacial meltwater controls the water mass properties and subsequent microbial responses in the Svalbard fjords (Rokkan Iversen and Seuthe 2011, Han et al. 2021, 2022). This study categorized the fjord waters into surface and subsurface layers according to water mass properties. The microbial diversity and community composition in these two layers were distinct. Unlike previous studies on eDNA metabarcoding in Arctic fjords, we investigated the interactions between phytoplankton and bacterial communities using comprehensive 16S and 18S rRNA gene metabarcoding. The 16S rRNA metabarcoding results suggested a homogenous community composition comprising a few dominant heterotrophic bacteria in the summer fjord waters. However, we found that the distribution of SAR11 clade Ia, specifically within the Alphaproteobacteria, reveals the subtle yet significant sample-to-sample variation within what initially appeared as a uniform bacterioplankton community. This finding suggests the complex interactions between microbial communities and their environments, indicating that taxa with widespread distribution, like Alphaproteobacteria, display considerable ecological variation. This variation highlights the adaptive capacity of these organisms across different environmental niches, reflecting their substantial role in ecosystem dynamics. Conversely, the diversity indices derived from the 18S rRNA data suggested a spatially diverse composition for eukaryotic communities.

Notably, phytoplankton populations within eukaryotic communities exhibited spatial variations in surface waters, whereas cyanobacteria, the photosynthetic population in bacterial communities, were dominant in surface waters. Although the relative abundance of heterotrophic bacteria varied from surface to subsurface waters, their widespread presence throughout the fjord waters indicates a robust heterotrophic component capable of utilizing a broad range of organic substrates, often originating from phytoplankton. This interplay between phytoplankton and heterotrophic bacteria suggests an ecological interface within the Arctic fjords. For example, the fate of phytoplankton-derived organic matter is linked to the biogeochemical processes mediated by heterotrophic bacterial metabolism during the oceanic nutrient cycle (Seymour et al. 2017).

DMSP, synthesized by marine plankton, is ubiquitous and is the most important source of biogenic sulfur and carbon in the ocean (Zindler et al. 2013). This phytoplankton-derived organic matter can be converted to substantial nutrients by heterotrophic bacterial activity. Indeed, the measured DMSP in the fjord waters suggests the existence of DMSP-rich phytoplankton in the summer surface waters of the Svalbard fjords. Moreover, we found that Dinophyceae is a predominant eukaryotic plankton in fjord waters, and such dominant phytoplankton have been considered one of the main producers of DMSP pools in the ocean (Keller et al. 1989, Steinke et al. 2002, Zindler et al. 2013).

We summarized a comparative description of DMSP in a microbial network model to understand the fate of phytoplankton-derived organic matter in fjord water. Microbial co-occurrence in the network model suggested biological interactions, such as competition, mutualism and predation, among the microbial communities in the Svalbard fjords. Microbial connections in the network represented the trophic role of the planktonic food web. For example, the link between Diatomea (phytoplankton) and Arthropoda (zooplankton) indicated their general feeding habits within plankton communities. Notably, a similar distribution of Dinophyceae (phytoplankton) and Intramacronucleata (zooplankton) was observed in the network model, with a strong linkage. Given that Intramacronucleata is a subphylum of ciliates that feed on small phytoplankton, such as Dinophyceae, the dominant Dinophyceae in the summer fjord waters may support the distribution of phytoplankton feeders, such as Intramacronucleata. Moreover, organic detritus products, such as DMSP, from plankton feeding habits, may provide nutrients for heterotrophic bacterial growth in the fjord waters. This assumption is supported by the qPCR measurement of bacterial DMSP degradation genes in summer Svalbard waters (Han et al. 2021).

We assumed that heterotrophic bacteria influenced DMSP metabolism in fjord waters. First, the homogeneous distribution of heterotrophic bacteria may frequently include DMSP in the fjord waters. Although the most dominant bacteria, such as Alphaproteobacteria, Gammaproteobacteria and Bacteroidia, were not linked to phytoplankton in the network model, these predominant heterotrophic bacteria may consume dissolved DMSP in the fjord waters after phytoplankton release. Nonetheless, less dominant bacteria, such as Verrucomicrobiae and Actinobacteria, were directly connected to phytoplankton (Prasinophytae or Dinophyceae) in the network model. As measured in this study, the DMSP concentration in natural seawater generally falls within the tens of nanomolar range (Kettle et al. 1999, Barak-Gavish et al. 2018). However, phytoplankton-derived organic matter is found at higher concentrations in the immediate surroundings of phytoplankton cells (phycosphere), where the organic matter released by the cell enhances the local environment of the surrounding water (Stocker 2012, Seymour et al. 2017). Mutualistic infochemical exchange within the phycosphere is an attractive strategy for recycling phytoplankton detritus, including DMSP, using heterotrophic bacteria. Indeed, marine Actinobacteria, connected to both Prasinophytae and Dinophyceae in the network, have been recognized for their significant involvement in the decomposition of cellulose, chitin, agar, laminarin, alginates and various hydrocarbons (Manivasagan et al. 2014), and increasing evidence suggests that Actinobacteria are involved in DMSP metabolism (Yoch et al. 2001, Mizuno et al. 2015, Liu et al. 2018). Therefore, understanding the interactions between phytoplankton and heterotrophic bacteria is essential for predicting the responses of ecological and biogeochemical processes in Arctic fjords and other marine environments.

Increasing information on phytoplankton-bacterial interactions has revealed a pattern of mutual exchange of substrates, such as essential vitamins and nutrients (Croft et al. 2005, Amin et al. 2009, Wang et al. 2014), and infochemicals, which convey information (Pohnert et al. 2007, Seyedsayamdost et al. 2011, Amin et al. 2015, Barak-Gavish et al. 2018), within the phycosphere (Segev et al. 2016, Landa et al. 2017). We predicted metabolic pathways for potential bacterial activity in phytoplankton-derived organic matter, including DMSP. Additionally, we assumed that the interactions between phytoplankton and bacteria could be estimated using the following metabolic pathways: sulfur metabolism (energy metabolism), benzoate degradation (xenobiotic biodegradation), terpenoid backbone biosynthesis (metabolism of terpenoids and polyketides), and penicillin and cephalosporin biosynthesis (biosynthesis of other secondary metabolites).

Hypothetical pathways for sulfur metabolism involved in bacterial DMSP and benzoate degradation were suggested in fjord waters. DMSP serves as a chemoattractant and facilitates bacterial interactions with phytoplankton. Phytoplankton-derived aromatic benzoate compounds promote bacterial growth, and bacterial interactions with phytoplankton are influenced by their capacity to utilize benzoate (Barak-Gavish et al. 2023). For example, the bacterial detection of DMSP, along with more specific compounds, such as benzoate, contributes to the recognition of phytoplankton hosts within the phycosphere by bacteria. Similarly, the biosynthesis of terpenoids (or terpenes) and β-lactam antibiotics (penicillin and cephalosporin) may provide a competitive advantage for phycosphere development. For example, terpenoids play diverse roles in mediating antagonistic and beneficial interactions among organisms against predators, pathogens and competitors, and convey messages to mutualists, signaling the presence of nutrients and potential threats (Gershenzon and Dudareva 2007). Although terpenoids are mainly produced by plants and fungi, some bacterial taxa in the phycosphere also synthesize terpenoid compounds (Lu et al. 2023). Moreover, synthesizing antibacterial compounds represents a mechanism through which a specific bacterial species can outcompete another, leading to enhanced and successful colonization linked with phytoplankton (Zhu et al. 2021).

Although our assumptions derived from this study provide meaningful information, certain limitations need careful consideration. First, this study focused on the post-blooming of phytoplankton in the summer season owing to the limitation of field observation. However, microbial interactions within summer may not represent the entire nutrient cycle of the Arctic fjords. Considering the microbial response to the pre-blooming in the spring season could provide a comprehensive understanding of the interaction between phytoplankton and bacterial communities in the fjords. Second, the influence of heterotrophic bacteria on DMSP metabolism is derived from the predicted metabolic functions. The prediction should be validated through the direct measurement of DMSP metabolism. Addressing these limitations could contribute to a robust and comprehensive understanding of the ecological and biogeochemical processes in the Arctic fjords. In addition, using rRNA gene regions for metabarcoding can lead to an overestimation of microbial community abundances, attributed to variations in rRNA gene copy numbers across different species. Species within microbial communities exhibit significant variation in the number of rRNA gene copies in their genomes. Consequently, taxa with higher gene copy numbers might be perceived as more abundant than they truly are in metabarcoding studies, as each gene copy is equally likely to be sequenced. This variation necessitates a cautious interpretation of metabarcoding data, acknowledging its semi-quantitative nature. Despite these challenges, metabarcoding is a powerful tool for exploring biodiversity and understanding ecological and biological processes, provided its limitations are recognized and appropriately addressed.

In conclusion, eDNA metabarcoding is a promising technique for studying phycosphere community composition in the Arctic fjord of Svalbard. Although recent microscopic observations have revealed the biogeography of phytoplankton and their contribution to the Arctic fjord food web, comprehensive studies validating the interactions between phytoplankton and bacterial communities in Arctic fjords are currently limited. Our results provide valuable insights into the responses of phycospheres to environmental change in this sensitive ecosystem. In particular, the metabolic pathways predicted by PICRUSt2 suggested a potential interaction between phytoplankton and heterotrophic bacteria. Our predicted metabolic processes may provide clues for seeking direct evidence of the role of the phycosphere in Arctic fjords using further deep sequencing, such as shotgun metagenome sequencing.

## Supplementary Material

fiae059_Supplemental_Files
