# Supplementary material for: Interaction between phytoplankton and heterotrophic bacteria in Arctic fjords during the glacial melting season as revealed by eDNA metabarcoding
Source: FEMS Microbiol Ecol. 2024 Apr 15;100(5):fiae059. doi: 10.1093/femsec/fiae059 (PMC11067963; doi:10.1093/femsec/fiae059)
Supplement: fiae059_Supplemental_Files [file fiae059_supplemental_files.zip › Supplementary data_Information (1).docx]

Interaction between Phytoplankton and Heterotrophic Bacteria in Arctic Fjords during Glacial Melting Season as revealed by eDNA Metabarcoding

Dukki Han^1^**^*^**, Ki-Tae Park^2,6^, Haryun Kim^3^, Tae-Hoon Kim^4^, Man-Ki Jeong^5^, and Seung-Il Nam^2^

^1^Department of Marine Molecular Bioscience, Gangneung-Wonju National University, 7, Jukheon-gil, Gangneung-si, Gangwon-do 25457, Republic of Korea

^2^Korea Polar Research Institute, Incheon 21990, Republic of Korea

^3^East Sea Research Institute, Korea Institute of Ocean Science & Technology, Uljin, 36315, Republic of Korea

^4^Department of Oceanography, Faculty of Earth Systems and Environmental Sciences, Chonnam National University, Gwangju, 61186, Republic of Korea

^5^Department of Smart Fisheries Resources Management, Chonnam National University, Daehak-ro 50, Yeosu 59626, Republic of Korea

^6^Department of Environmental Sciences and Biotechnology, Hallym University, Chuncheon, Gangwon-do, 24252, Republic of Korea

**^*^Corresponding author:** Dukki Han (dukkihan@gwnu.ac.kr)


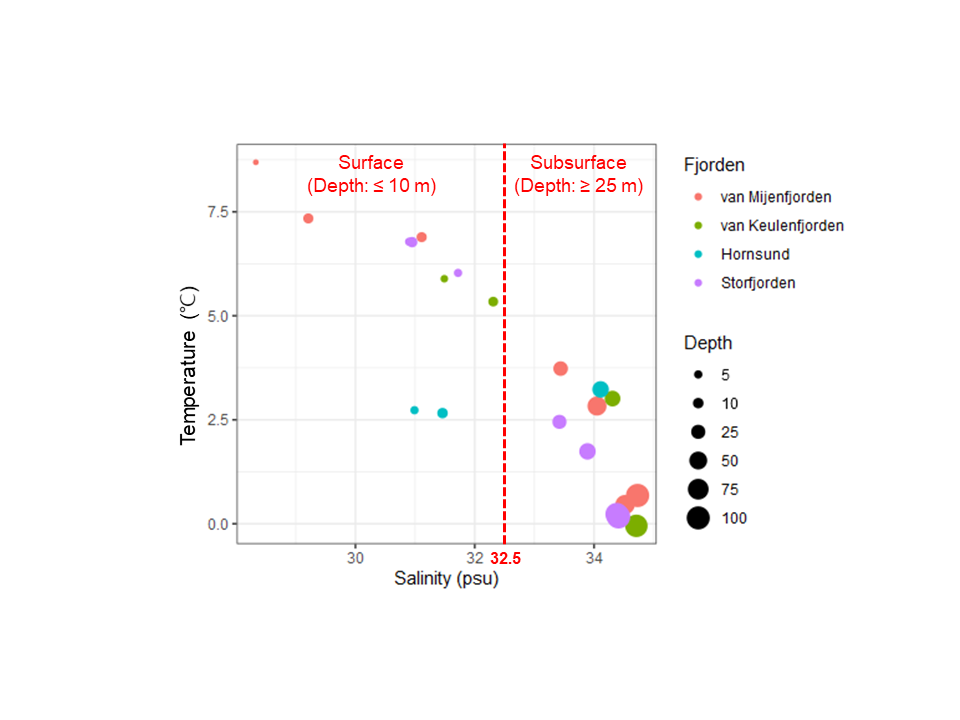


Figure S1. Temperature and salinity diagram in fjord waters.


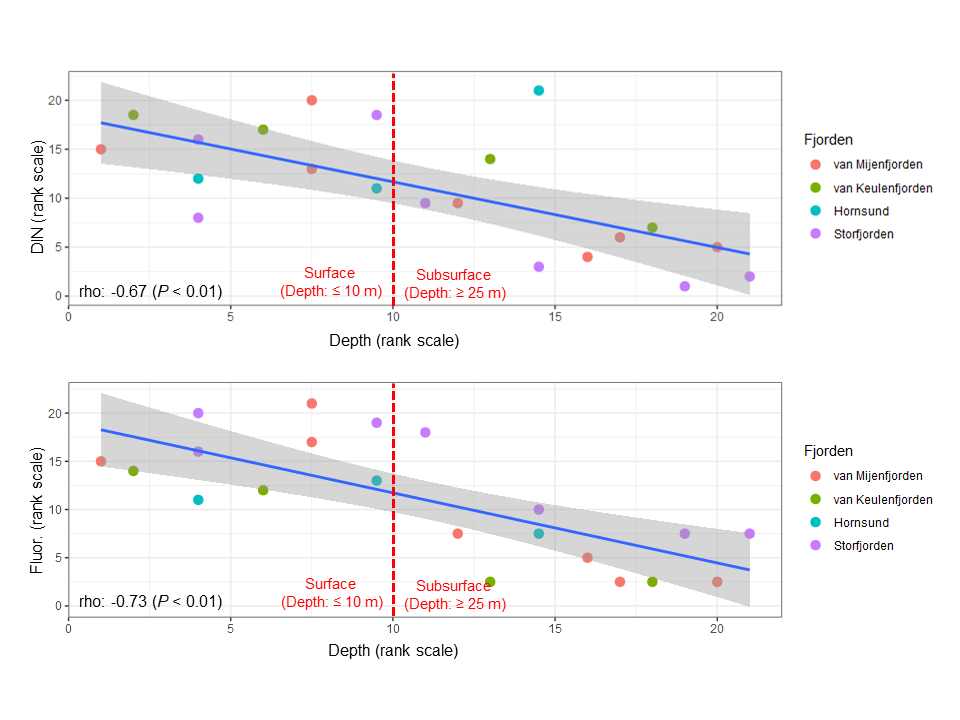


Figure S2. Distribution of DIN and chlorophyll fluorescence at depth change.


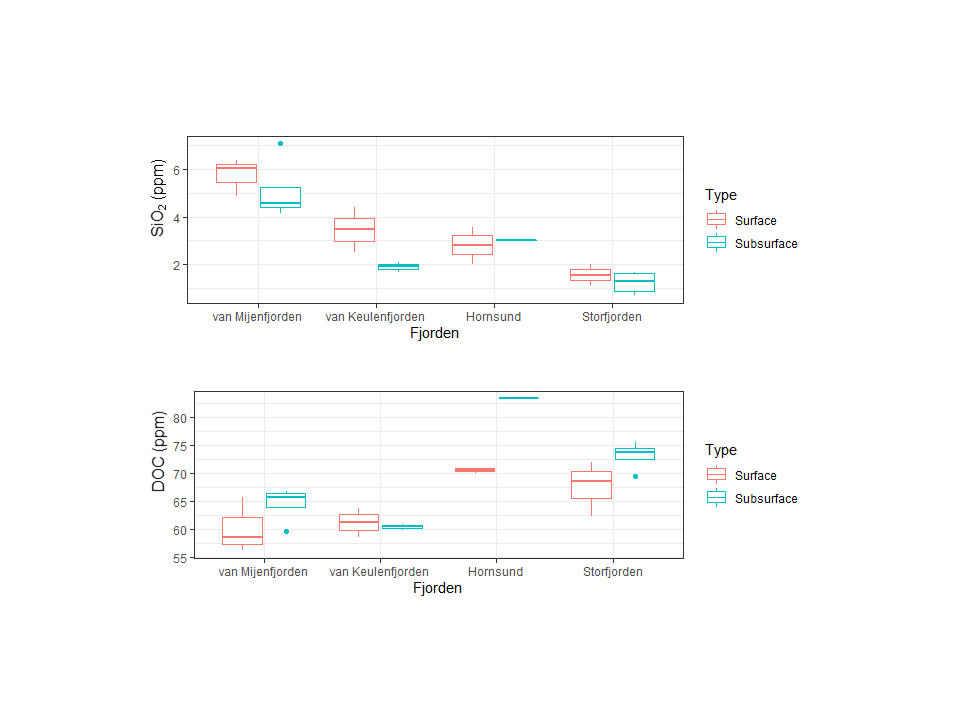


Figure S3. Variation of SiO_2_ and DOC concentrations in each fjord.


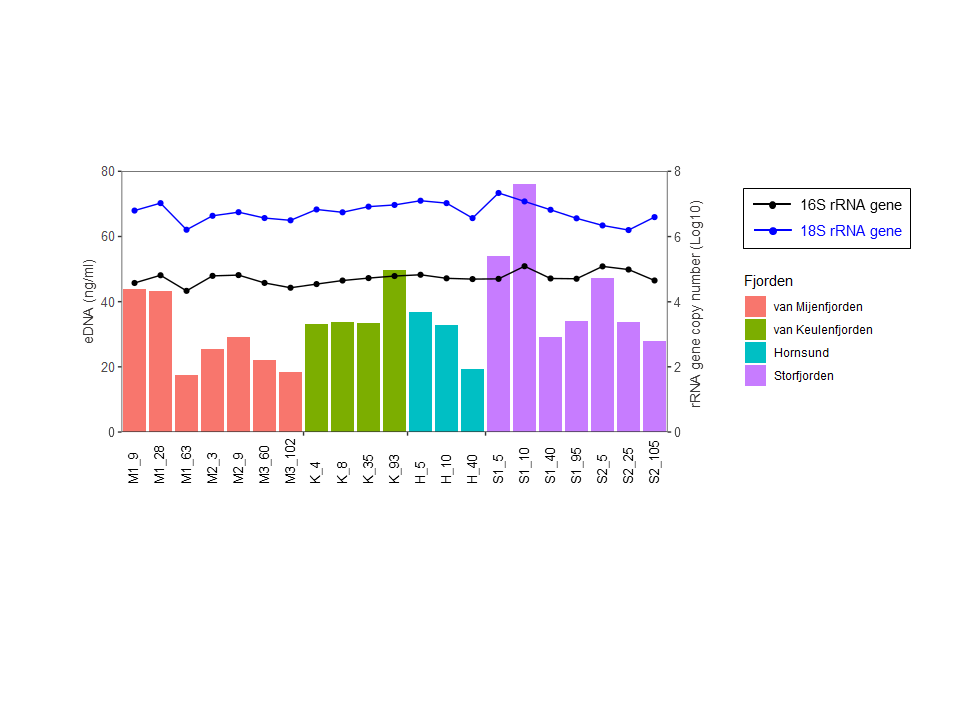


Figure S4. Distribution of eDNA concentration (bar plot) and rRNA copy number (line plot) in fjord waters. Concentration of eDNAs was significantly correlated with copy numbers of 16S (Cor > +0.5, *P* < 0.05) and 18S (Cor > +0.5, *P* < 0.05) rRNA genes, respectively. There was no significant correlation between copy numbers of 16S and 18S rRNA genes (Cor < +0.5, *P* > 0.05).


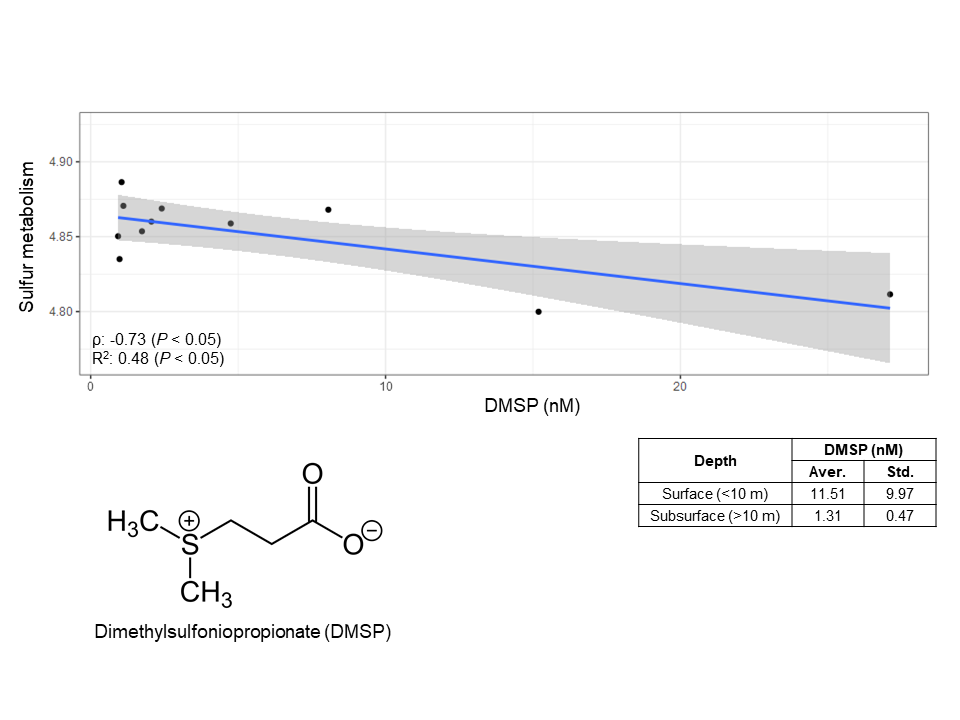


Figure S5. Comparison of DMSP concentration with the predicted sulfur metabolism.


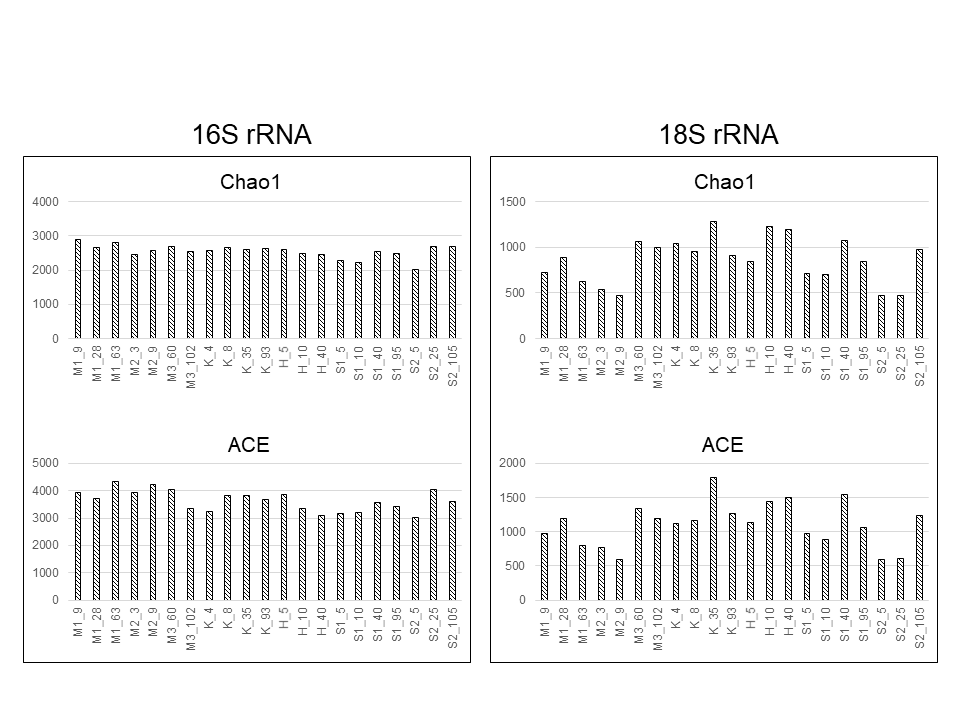


Figure S6. Distribution of alpha diversity indices in fjord waters.


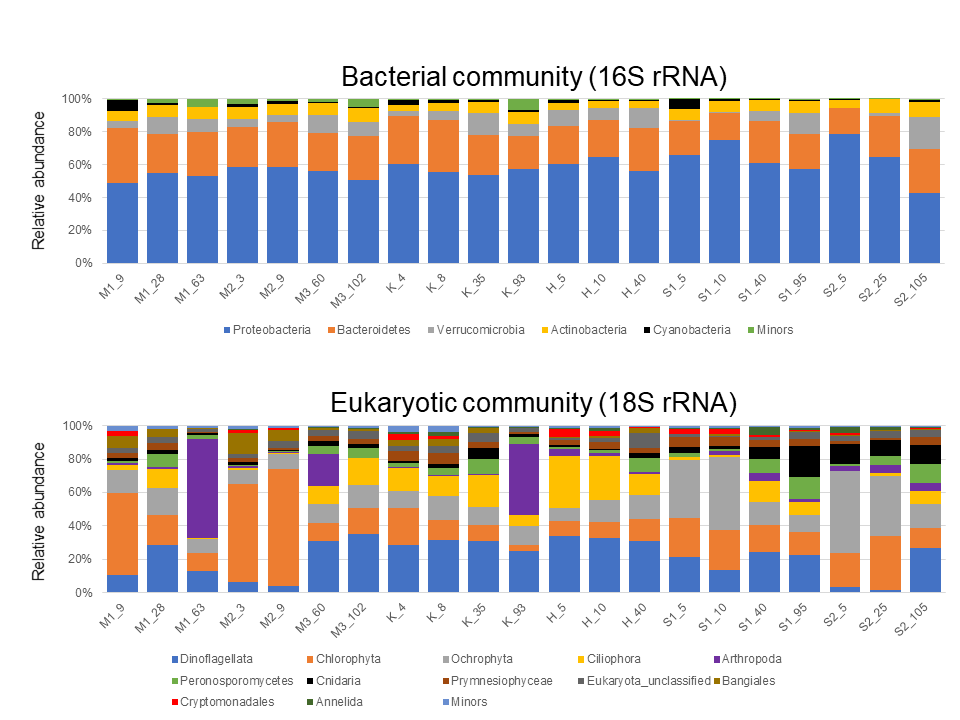


Figure S7. Bacterial and eukaryotic community compositions at phylum level.


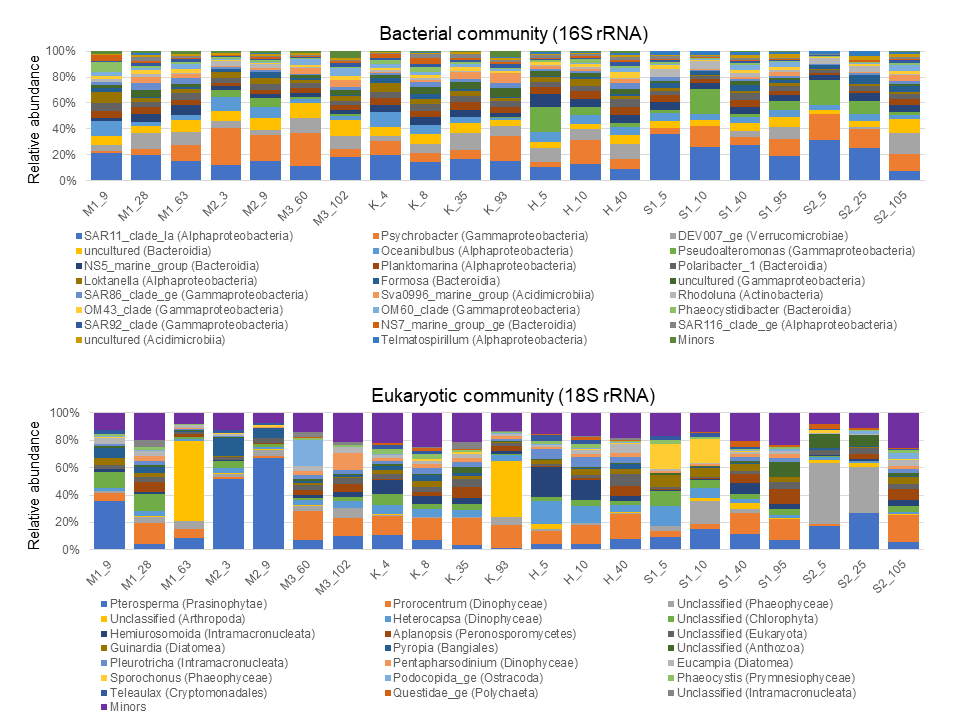


Figure S8. Bacterial and eukaryotic community compositions at genus level.


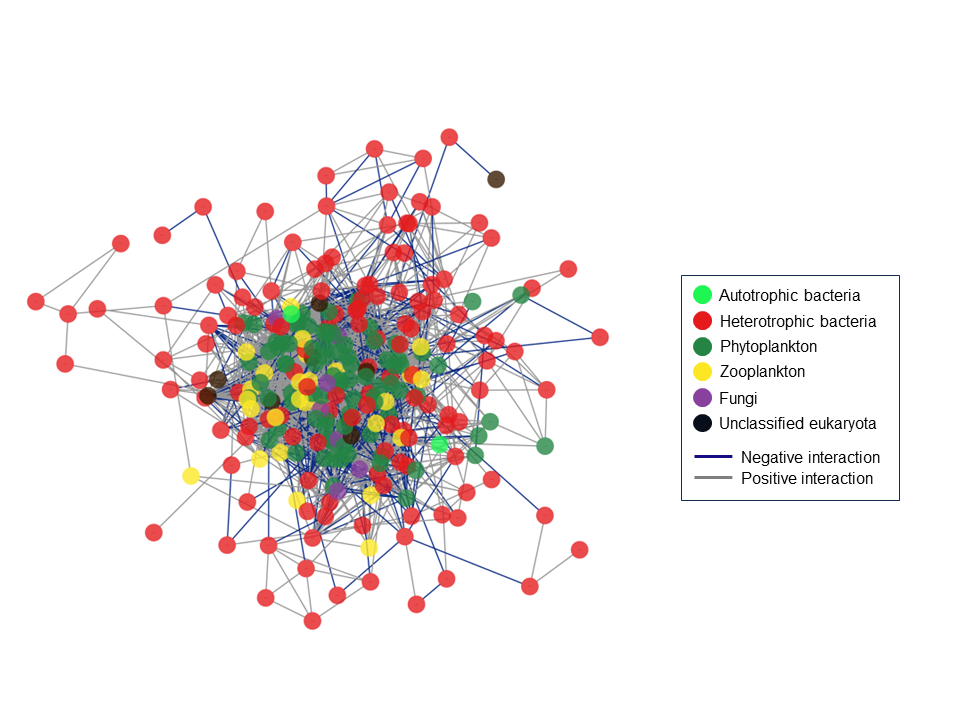


Figure S9. Microbial network model based on the ASVs. In the network model, each node indicates ASVs, and edges of gray lines and blue lines represent positive and negative correlations at significant level (correlation coefficient > 0.50, *P* < 0.05), respectively.


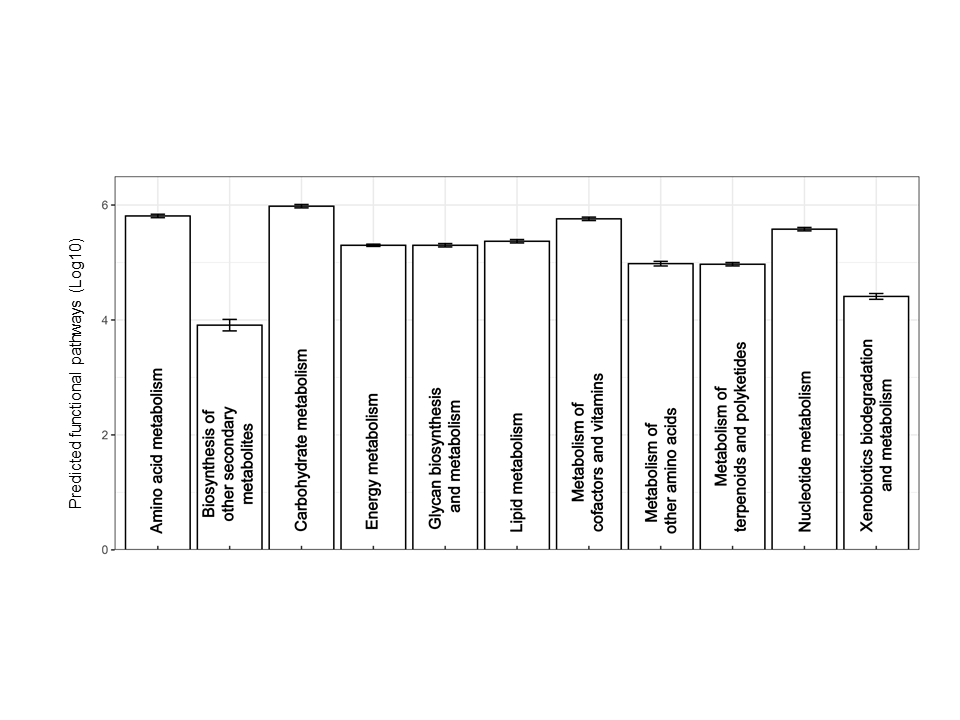


Figure S10. Prediction of bacterial metabolism by PICRUSt2


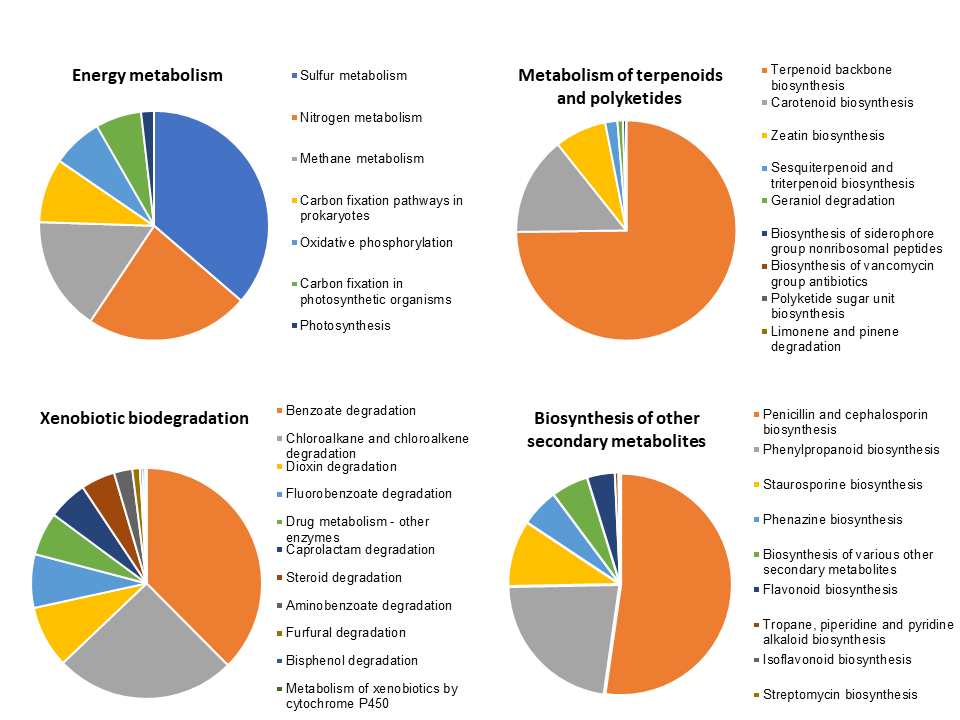


Figure S11. Associated bacterial pathways in energy metabolism, xenobiotic biodegradation, metabolism of terpenoids and polyketides, and biosynthesis of secondary metabolites

Table S1. Statistical separation for 16S and 18S rRNA gene data supported by AMOVA. Significant differences within group or between pairs were marked in bold and underlined (*P* < 0.01).

| AMOVA | *P*-value | |
| --- | --- | --- |
|  | 16S rRNA | 18S rRNA |
| Hornsund-Keulenfjorden-Mijenfjorden-Storfjorden | > 0.01 | **< 0.01** |
| Hornsund-van Keulenfjorden | > 0.01 | > 0.01 |
| Hornsund-van Mijenfjorden | > 0.01 | > 0.01 |
| Hornsund-Storfjorden | > 0.01 | > 0.01 |
| van Keulenfjorden-van Mijenfjorden | > 0.01 | > 0.01 |
| van Keulenfjorden-Storfjorden | > 0.01 | > 0.01 |
| van Mijenfjorden-Storfjorden | > 0.01 | **< 0.01** |
| Surface-Subsurface | **< 0.01** | **< 0.01** |

Table S2. Indicator species analysis for 16S and 18S rRNA data in each fjord. The significant values (*P* < 0.01) were highlighted in bold and underlined.

| rRNA | Taxa (Class) | Fjorden | Indicator value | *P*-value |
| --- | --- | --- | --- | --- |
| 16S | Oxyphotobacteria | van Mijenfjorden | 0.32 | > 0.01 |
|  | Bacteroidia | van Mijenfjorden | 0.27 | > 0.01 |
|  | Acidimicrobiia | van Keulenfjorden | 0.32 | > 0.01 |
|  | Verrucomicrobiae | Hornsund | 0.33 | > 0.01 |
|  | Gammaproteobacteria | Hornsund | 0.28 | > 0.01 |
|  | Actinobacteria | Storfjorden | 0.39 | **< 0.01** |
|  | Alphaproteobacteria | Storfjorden | 0.28 | > 0.01 |
| 18S | Ostracoda | van Mijenfjorden | 0.53 | > 0.01 |
|  | Bangiales_cl | van Mijenfjorden | 0.51 | > 0.01 |
|  | Prasinophytae | van Mijenfjorden | 0.48 | > 0.01 |
|  | Chlorophyta_unclassified | van Mijenfjorden | 0.30 | > 0.01 |
|  | Arthropoda_unclassified | van Keulenfjorden | 0.47 | > 0.01 |
|  | Prymnesiophyceae_cl | van Keulenfjorden | 0.34 | > 0.01 |
|  | Chrysophyceae | van Keulenfjorden | 0.33 | > 0.01 |
|  | Intramacronucleata | Hornsund | 0.50 | > 0.01 |
|  | Cryptomonadales_cl | Hornsund | 0.42 | > 0.01 |
|  | Dinophyceae | Hornsund | 0.34 | > 0.01 |
|  | Diatomea | Hornsund | 0.31 | > 0.01 |
|  | Eukaryota_unclassified | Hornsund | 0.29 | > 0.01 |
|  | Phaeophyceae | Storfjorden | 0.67 | > 0.01 |
|  | Anthozoa | Storfjorden | 0.58 | **< 0.01** |
|  | Polychaeta | Storfjorden | 0.45 | > 0.01 |
|  | Prymnesiales | Storfjorden | 0.34 | > 0.01 |
|  | Peronosporomycetes | Storfjorden | 0.34 | > 0.01 |
